# Myelodysplastic Syndrome in Pakistan: Clinicohematological Characteristics, Cytogenetic Profile, and Risk Stratification

**DOI:** 10.4274/tjh.2017.0130

**Published:** 2018-05-25

**Authors:** Rafia Mahmood, Chaudry Altaf, Parvez Ahmed, Saleem Ahmed Khan, Hamid Saeed Malik

**Affiliations:** 1 Armed Forces Institute of Pathology, Department of Hematology, Rawalpindi, Pakistan

**Keywords:** Myelodysplastic syndrome, Cytogenetics, Revised International Prognostic Scoring System

## Abstract

**Objective:**

Myelodysplastic syndrome (MDS) is a group of bone marrow diseases that not only have variable morphological presentation and heterogeneous clinical courses but also have a wide range of cytogenetic abnormalities. Clinicohematological parameters have a significant role in diagnosis and along with identification of cytogenetic abnormalities are important for prognostic scoring and risk stratification of patients to plan management and make treatment decisions. This study aimed to determine the clinicohematological characteristics, cytogenetic abnormalities, and risk stratification of newly diagnosed de novo MDS patients.

**Materials and Methods:**

This cross-sectional study was conducted in the Department of Hematology, Armed Forces Institute of Pathology, Rawalpindi, from January 2013 to January 2017. Patients were diagnosed on the basis of World Health Organization criteria for MDS, clinicohematological parameters were noted, and cytogenetic analysis was performed. Risk stratification was done using the Revised International Prognostic Scoring System.

**Results:**

A total of 178 cases of MDS were analyzed, including 119 males (66.9%) and 59 females (33.1%). The median age was 58 years. The most common presenting feature was anemia in 162 (91%) of the patients. MDS with multilineage dysplasia was the most common diagnosis, seen in 103 (57.9%) patients. A normal karyotype was seen in 95 (53.4%), while 83 (46.6%) showed clonal karyotypic abnormalities at diagnosis. Of these, the common abnormalities found were trisomy 8, complex karyotype, and del 5q. Risk stratification revealed low-risk disease in 73 (41%) patients.

**Conclusion:**

Cytogenetic analysis showed the normal karyotype to be the most common while risk stratification revealed a predominance of low-risk disease at the time of presentation.

## Abstract (tr)

**Amaç:**

Myelodisplastik sendrom (MDS) sadece değişken morfolojik prezentasyona ve heterojen klinik seyre değil geniş sitogenetik anormallikler yelpazesine de sahip olan bir grup kemik iliği hastalığıdır. Klinikohematolojik parametreler tanıda önemli role sahiptir ve sitogenetik anormalliklerin tanımlanması ile birlikte prognostik skorlamada ve yönetimi planlamak ve tedavi kararlarını vermek için risk stratifikasyonunda önemlidir. Bu çalışma, yeni tanı de novo MDS hastalarında klinikohematolojik özellikler, sitogenetik anormallikler ve risk stratifikasyonunu belirlemeyi amaçlamıştır.

**Gereç ve Yöntemler:**

Bu kesitsel çalışma Rawalpindi Silahlı Kuvvetler Patoloji Enstitüsü Hematoloji Departmanı’nda Ocak 2013’ten Ocak 2017 tarihine kadar sürdürülmüştür. Hastalar Dünya Sağlık Örgütü MDS kriterlerine göre teşhis edildi, klinikohematolojik parametreler not edildi ve sitogenetik analiz yapıldı. Risk stratifikasyonu Revize Uluslararası Prognostik Skorlama Sistemi kullanılarak yapıldı.

**Bulgular:**

Toplam 178 MDS olgusu, 119 erkek (%66,9) ve 59 kadın (%33,1) analiz edildi. Medyan yaş 58 idi. Başvuruda en sık görülen belirti olguların 162’sinde (%91) anemi idi. En sık tanı MDS çoklu seride displazi olup 103 (%57,9) hastada görüldü. Teşhiste normal karyotip 95 (%53,4) olguda görülürken 83 (%46,6) olgu klonal karyotipik anormallikler gösterdi. Bunlar arasında, en sık görülenler trizomi sekiz, kompleks karyotip ve del5q idi. Risk stratifikasyonu 73 (%41) hastada düşük-risk hastalık ortaya koydu.

**Sonuç:**

Sitogenetik analiz en sık normal karyotipi gösterirken risk stratifikasyonu tanı sırasında düşük-risk hastalığın çoğunlukta olduğunu ortaya koymuştur.

## Introduction

Myelodysplastic syndrome (MDS) is a heterogeneous group of clonal stem cell disorders characterized by peripheral blood cytopenias, dysplasia, and ineffective hematopoiesis [1]. Patients have a variable clinical course and there is an increased risk of myeloid leukemic transformation [2]. It is a disease of the elderly; its incidence increases with age. It is slightly more common in males with a male:female ratio of 1.4:1 [3]. While a few patients may be detected incidentally when a routine blood count reveals unexpected cytopenia, most present with symptoms and signs of bone marrow failure. Notable findings include fatigue due to anemia, infections, and bleeding [4].

Morphologic dysplasia is the hallmark of the disease [5]. Dysplasia may be seen in any or all of the three lineages [6]. The World Health Organization (WHO) has classified the myelodysplastic syndromes based on the number of cytopenias, dysplasia in a single lineage or in multiple lineages, cytogenetics, the number of blast cells, and the presence or absence of ring sideroblasts [5].

In addition to morphologic heterogeneity, MDS cases show profound heterogeneity in their genetic presentation [7]. More than half the patients show clonal chromosomal abnormalities with a predominance of unbalanced abnormalities [8]. Cytogenetic analysis not only has an important role in diagnosis where certain chromosomal abnormalities are considered presumptive evidence of MDS, but also has important prognostic implications. These cytogenetic abnormalities can be detected by conventional metaphase karyotyping. However, fluorescent in situ hybridization (FISH) has been seen to have a much higher sensitivity for detection of del 5q [9]. These cytogenetic findings serve as a basis for the characterization of cytogenetic subgroups [10].

Over time, a better understanding of the biology of disease has shown cytogenetics to be an important prognostic parameter [11]. The Revised International Prognostic Scoring System (R-IPSS) refines risk group definitions, aiming for better prediction of individual prognosis. The parameters included are the degree of cytopenias, the number of blast cells, and the cytogenetic subgroup [12]. The prognostication of patients based on individualized risk assessment not only predicts disease progression but also provides an important tool in planning management and making treatment decisions [13].

Most studies regarding MDS are from Western populations. Disease biology, clinical presentations, and cytogenetic findings are different and distinctive for population groups and can show noticeable differences in geographic prevalence around the world. The present study was designed with an aim to see the clinicohematological features, cytogenetic profile, and risk stratification of the patients of Pakistan (an Asian population) as so far there is a lack of data on MDS in our region. This will help to determine treatment protocols and prognosis.

## Materials and Methods

### Patients

This study was a cross-sectional analysis conducted in the Department of Hematology of the Armed Forces Institute of Pathology, Rawalpindi, from January 2013 to January 2017. All patients were Pakistanis, of Asian origin, belonging to different ethnic groups including Punjabis, Pashtuns, Sindhis, Balochis, Kashmiris, and those from Gilgit-Baltistan. Patients were between the ages of 30 and 85 years. These patients were newly diagnosed with MDS and had no previous history of any treatment. Patients who had failed culture (did not yield at least 20 metaphases) in cytogenetic analysis were excluded from the study. All subjects were thoroughly informed about the study and written informed consent was obtained.

### Clinicohematological Parameters

Detailed history was recorded and complete physical examination was done. Symptoms and signs were noted. Complete blood count, peripheral blood film, and bone marrow examination were done and patients were diagnosed as having MDS based on the WHO criteria.

### Cytogenetics and FISH

Cytogenetic analysis was performed by using the conventional G banding technique. A bone marrow specimen of 3 mL was collected in sodium heparin. Metaphase chromosomes were banded using the conventional Giemsa trypsin banding technique and karyotyped according to the International System for Human Cytogenetic Nomenclature criteria. At least twenty metaphases were analyzed with the CytoVision semiautomated image analysis and capture system.

Interphase FISH studies were performed on blood or bone marrow specimens processed by standard methods for cultured samples. The MetaSystems XL 5q31/5q33 probe (10 µL) was applied to the target on the slide. A total of 500 nuclei were analyzed per probe set by using a fluorescent microscope with an orange green spectrum filter.

### Risk Stratification

The patients were risk-stratified according to the R-IPSS.

### Statistical Analysis

Collected data were entered and analyzed using SPSS 20 (IBM Corp., Armonk, NY, USA). Quantitative variables, i.e. age, hemoglobin (Hb), platelet count, and absolute neutrophil count (ANC), have been presented as mean ± standard deviation. Qualitative variables, i.e. sex, cytogenetics, and risk category, have been presented as frequency and percentage.

### Ethical Approval

This study was approved by the Ethical Review Committee of the Armed Forces Institute of Pathology, Rawalpindi. Informed written consent was received from the patients.

## Results

A total of 178 patients were diagnosed as having de novo MDS. The median age of the patients was 58 years. Out of 178 patients, 119 (66.9%) were male, while the remaining 59 (33.1%) patients were female.

The most common presenting clinical feature was pallor, followed by symptoms of fatigue, recurrent infections, and bruising/bleeding; 118 (66%) of the patients were transfusion-dependent at the time of presentation. Mean Hb was 6.4 g/dL and mean platelet count was 97x10^9^/L, while the mean ANC was 2.1x10^9^/L. Table 1 shows the clinicohematological parameters of our patients. We classified our patients according to the 2016 revised WHO classification: 103 (57.9%) of the patients were in the MDS-MLD (MDS with multilineage dysplasia) category while 36 (20.2%) cases were classified as MDS-SLD (MDS with single lineage dysplasia), 16 (8.9%) as MDS-EB1 (MDS with excess blasts-1), 12 (6.7%) as MDS-EB2 (MDS with excess blasts-2), 6 (3.4%) as MDS with isolated del (5q), 3 (1.7%) as MDS-RS-SLD (MDS with ring sideroblasts with single lineage dysplasia), and 2 (1.1%) as MDS-RS-MLD (MDS with ring sideroblasts with multilineage dysplasia).

**Figure 1:**
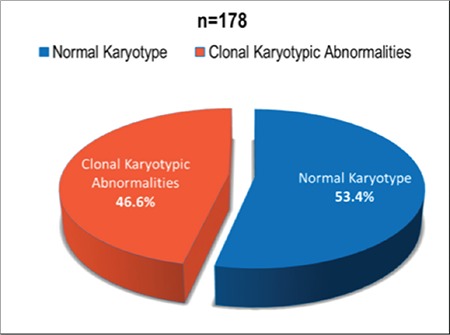
Cytogenetics of the patients.

**Figure 2:**
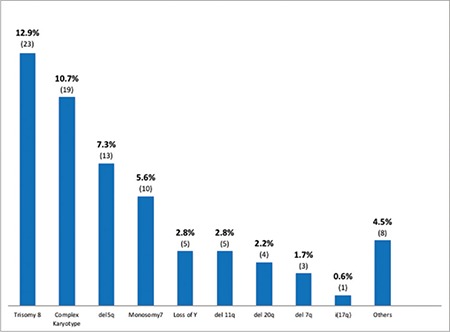
Common karyotypic abnormalities of the patients.

A normal karyotype was seen in 95 (53.4%) cases, while 83 (46.6%) patients showed clonal karyotypic abnormalities at diagnosis (Figure 1 and 2). Of these, 56 (31.4%) had single and 8 (4.5%) had double cytogenetic abnormalities while 19 (10.7%) had a complex karyotype. Of the cytogenetic abnormalities seen, the most commonly found was trisomy 8 in 23 (12.9%) cases, followed by del 5q in 13 (7.3%), monosomy 7 in 10 (5.6%), loss of Y in 5 (2.8%), del 11q in 5 (2.8%), del 20q in 4 (2.2%), del 7q in 3 (1.7%) and i(17q) in 1 (0.6%) patient. Other abnormalities, including translocations, hyperdiploidy, hypodiploidy, deletions, and monosomies, were seen in 8 (4.5%) of the patients. del 5q was detected in 8 patients based on conventional cytogenetics while in 5 patients it was missed by conventional cytogenetics and detected by FISH.

Each parameter was assessed and scored according to the R-IPSS. Based on the score, the patients were stratified into five distinct risk groups. In the very-low-risk group, there were 17 (9.6%) patients, while there were 73 (41%) patients in the low-risk group, 48 (27.1%) patients in the intermediate-risk group, 24 (13.5%) patients in the high-risk group, and 16 (9.1%) patients in the very-high-risk group.

## Discussion

Myelodysplastic syndromes show not only clinical heterogeneity and genetic diversity but also a highly variable clinical course [14]. Over the last decade a better understanding of the biology of MDS has led to the identification of genetic molecular factors that have diagnostic value as well as roles in determining the disease course and prognosis [15]. Conventional cytogenetics of all newly diagnosed MDS patients is thus of paramount significance as it is an important component in risk-stratifying the patients [16]. FISH has a much higher sensitivity and has improved the detection of genomic aberrations in MDS, especially del 5q [9].

To our knowledge, there are no comprehensive data available on the clinicohematological features, cytogenetic profiles, and risk stratification of MDS patients from our part of the country. The Armed Forces Institute of Pathology is a tertiary care institute and a referral center in the north of Pakistan. It caters to a large number of patients from all over the country from very different ethnic backgrounds. Our study aims to help clinicians in structuring treatment decisions in light of cytogenetically based risk stratification.

In our study, the median age of the patients was 58 years. Similar findings have been reported in local studies. However, a much higher age, 71 years, was reported by Greenberg et al. [11] in a Western population. There is a major difference in the age of presentation of our patients and the Western population. These differences may be attributable to racial and geographic differences and differences in disease biology in different populations. Among our patients, males were more common as compared to females (66.9% vs. 33.1%). The male-to-female ratio was 2:1. Our observation coincides with the findings of Sultan and Irfan [17], who reported a sex ratio of 1.6:1. Deeg et al. [18] also reported a male predominance.

The most common presenting clinical findings of pallor followed by fatigue observed in our study are consistent with those reported by Narayanan [19] in the Indian population. Of our patients, 66% were transfusion-dependent at the time of presentation. A similar frequency of 58% was reported in the Italian population, while Greenberg et al. [11] reported 32% of the patients to be transfusion-dependent based on data from eleven countries. Mean Hb was 6.4 g/dL. Chaubey et al. [20] demonstrated mean Hb of 6.8 g/dL, which is in accordance with our findings. In another study, Voso et al. [12] reported mean Hb of 9.9 g/dL in Italian patients. There is a striking difference in presenting Hb levels and transfusion dependency in our population as compared to the Western populations studied by Greenberg et al. [11]. This may be due to the fact that Pakistan is a developing country and patients present late as they do not have early access to tertiary care medical facilities. However, these differences in presentation, with more than two-thirds of our patients being transfusion-dependent, may affect the overall treatment plan. These patients need further stratification by evaluation of their erythropoietin levels, which will guide further management. In patients with low erythropoietin levels (less than 200 IU/L), early institution of erythropoietin therapy predicts the response. Erythropoietin therapy not only improves Hb levels but also enhances the quality of life without the risks associated with blood transfusions. Iron chelation will also be an important consideration in patients who have received multiple transfusions.

In our study, the mean platelet count was 97x10^9^/L, while a mean platelet count of 100.5x10^9^/L was reported in Indians [20] and 152x10^9^/L [12] in the Italian population. The mean ANC in our study population was (2.1±1.8)x10^9^/L, which correlates with the median ANC of 1.9x10^9^/L reported by Voso et al. [12]. The clinicohematological characteristics of our study population are compared with those of national and international studies in Table 2.

On cytogenetic analysis, a normal karyotype was seen in 95 patients (53.4%), while 83 (46.6%) patients showed clonal karyotypic abnormalities at diagnosis. Chromosomal abnormalities were detected in 34.6% of cases by Narayanan [19], 39% by Voso et al. [12], 42.3% by Rashid et al. [21], 47.5% by Chaubey et al. [20], and 48% by Cao et al. [9]. A complex karyotype, which carries poor overall survival, was seen in 10.7% of our patients, while Rashid et al. [21] reported a frequency of 15.5%.

Table 3 shows a comparison of the cytogenetic profile with national and international data. In our study, the most common cytogenetic abnormality was trisomy 8 in 12.9% followed by del 5q in 7.3% and monosomy 7 in 5.6% of the patients. Rashid et al. [21] reported trisomy 8 to be the most common cytogenetic abnormality with a frequency of 9.9%. However, they reported a much lower frequency of del 5q in 2.8% of the patients. This difference may be due to the difference in the cytogenetic methodology adopted, as we used FISH for detection of del 5q in addition to conventional cytogenetics, as FISH has higher sensitivity. Chaubey et al. [20] reported monosomy 7 as the most frequent cytogenetic abnormality detected in 15%, followed by del 5q in 10% and trisomy 8 in 7.5% of Indian patients. In the Italian population [12], the most common karyotypic abnormality reported is del 5q in 10.5%, while much lower frequencies of 5% for trisomy 8 and 2% for monosomy 7 have been reported. Identification of patients with del 5q is particularly important as these patients are candidates for treatment with the immunomodulatory drug lenalidomide. Early initiation of treatment with lenalidomide not only leads to transfusion independence but also induces cytogenetic remission in this subgroup of patients. 

The R-IPSS score is particularly useful in clinical decision-making and selection of appropriate treatment options while at the same time providing prognostic information and predicting outcome in response to disease-modifying therapies. Upon risk stratification by the R-IPSS, as shown in Table 4, most of our patients (41%) were in the low-risk category, followed by 27.1% of the patients in the intermediate-risk category. These findings are in accordance with the findings of Greenberg et al. [11], who reported 38% of their patients in the low-risk followed by 20% in the intermediate-risk and 19% in the very-low-risk category. However, in an Italian study, Voso et al. [12] reported 38% in the very-low-risk category, followed by 33% in the low-risk and 18% in the intermediate-risk category. Those patients in the high-risk and very-high-risk groups need stringent regular monitoring as they have poor prognosis and are potentially more likely to have disease progression and transformation into acute myeloid leukemia.

## Conclusion

Cytogenetic analysis showed the normal karyotype to be the most common while among the cytogenetic abnormalities detected trisomy 8 was the most common. Risk stratification revealed a predominance of low-risk disease at the time of presentation. The results of our study are in accordance with other local studies with a few differences, which may be due to differences in the method of detection of chromosomal abnormalities. However, there are differences with studies in other parts of the world. These differences may be attributable to geographical and ethnic differences in disease biology and genetics. As MDS has a heterogeneous clinical course, genetic characterization of all newly diagnosed MDS patients is important not only for diagnosis but also for risk stratification so that individualized treatment can be instituted to improve survival and for predicting outcome.

## Figures and Tables

**Table 1:**
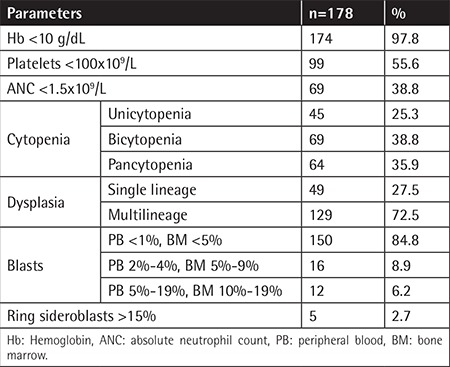
Clinicohematological parameters of the patients.

**Table 2:**
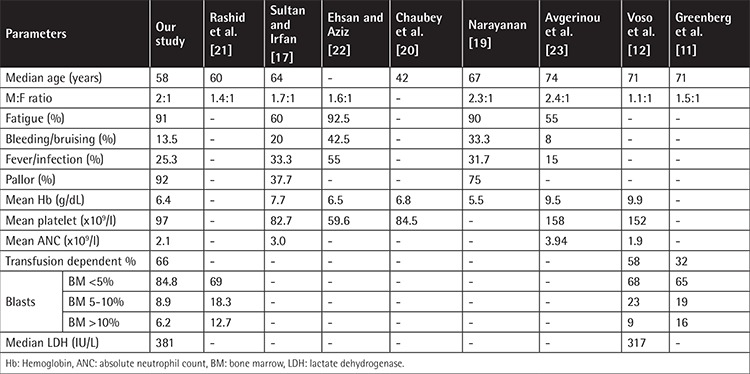
Comparison of clinicohematological characteristics with national and international studies.

**Table 3:**
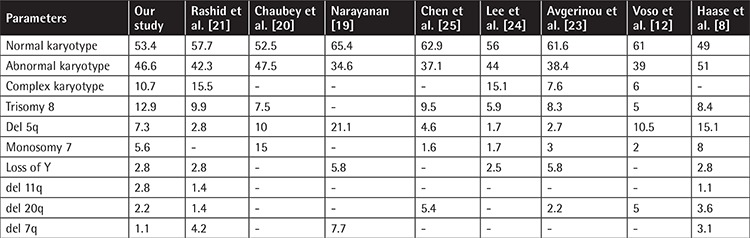
Cytogenetic profile in comparison with national and international studies.

**Table 4:**
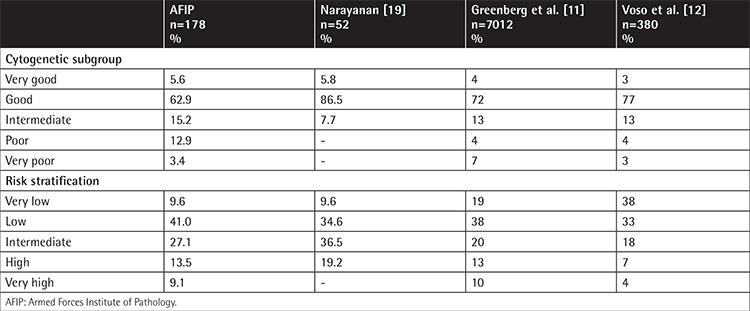
Comparison of cytogenetic subgroups and risk stratification.
